# Kidney dysfunction and cerebral microbleeds in neurologically healthy adults

**DOI:** 10.1371/journal.pone.0172210

**Published:** 2017-02-16

**Authors:** Sang Hyuck Kim, Dong Wook Shin, Jae Moon Yun, Ji Eun Lee, Jae-Sung Lim, Be Long Cho, Hyung-Min Kwon, Jin-Ho Park

**Affiliations:** 1 Department of Family Medicine, Seoul National University Hospital, Seoul, Republic of Korea; 2 Department of Neurology, Hallym University Sacred Heart Hospital, Anyang, Republic of Korea; 3 Department of Neurology, Seoul National University-Seoul Municipal Government Boramae Medical Center, Seoul, Republic of Korea; The University of Tokyo, JAPAN

## Abstract

**Introduction:**

Cerebral microbleed (CMB) is a potent risk factor for overt cerebrovascular disease. Although some studies indicated the possible role of renal dysfunction as a risk factor of CMB, the findings could not be generalized. This study aimed to investigate the association between renal dysfunction and cerebral microbleed (CMB) in neurologically healthy adults.

**Materials and methods:**

A total of 2,518 subjects who underwent brain MRI as part of health screening were involved in the study. CMBs were defined as well-demarcated focal areas of low signal intensity with associated blooming on the T2-weighted MRI measuring less than 5mm in diameter. The glomerular filtration rate (GFR) was estimated using the Modification of Diet in Renal Disease formula. Kidney function was classified as normal (≥90), mild (60 to 89.9), moderate (30 to 59.9), and severe (<30 mL/min/1.73 m^2^) renal dysfunction according to the GFR.

**Results:**

The mean age of the participants was 57.5 ± 8.3 years (ranged 40 to 79), and 1,367 subjects (54.3%) were male. The mean GFR level was 81.5 ± 15.5, and the prevalence of CMB was 4.1% (n = 103). Subjects with CMB demonstrated a higher proportion of moderate-to-severe renal dysfunction than those without CMB (15.5% vs. 5.0%, p < 0.001). In the multivariate logistic regression analysis, moderate-to-severe renal dysfunction showed a significant association with CMB (adjusted odd ratio = 2.63; *p* = 0.008). Furthermore, a decrease in the GFR level was associated with an increasing trend of the presence of CMB (*p* for trend = 0.031) and number of CMB lesions (*p* for trend = 0.003).

**Conclusions:**

Renal dysfunction was significantly associated with the presence of CMB in neurologically healthy adults. More studies are needed to evaluate if treatment of kidney disease and risk factor modification may prevent further progress of CMB.

## Introduction

Cerebral small vessel disease refers to various pathological processes of the cerebral vasculature that result in endothelial impairment and dysfunction.[1] Cerebral small vessel diseases, including cerebral microbleed (CMB), have recently attracted attention as they are potent risk factors for overt cerebrovascular disease (CVD), such as ischemic and hemorrhagic stroke.[1–3] CMB is an incidental finding from magnetic resonance imaging (MRI) in patients with focal neurologic deficit or even in healthy adults.[4] When compared to healthy adults, in patients with intracerebral hemorrhage (ICH), the prevalence of CMB is almost 10 times higher, even sharing an etiologic basis with intracerebral hemorrhage.[5] In addition, CMB is associated with cognitive impairment and Alzheimer’s disease.[6–9] Moreover, CMB is a potential predictor of symptomatic ICH recurrence,[10, 11] impairment in independence, and mortality in primary ICH patients.[12]

The clinical implications of CMB have led to concerns about identifying its risk factors.[13–16] Some studies focused on the association between renal dysfunction and CMB. They found that CMB was more frequently observed in subjects with than in those without renal dysfunction.[17–20] Although those studies suggested the possible role of renal dysfunction as a risk factor of CMB, the findings could not be generalized owing to critical limitations such as participants already experiencing advanced renal failure[19] or stroke,[17, 18, 20] extremely elderly population,[17, 18] population with a higher prevalence of other risk factors such as hypertension,[17, 18] subjects admitted to the hospital,[17, 18] and small study population, ranging from 97 to 186 individuals.[17–20]

We recruited a neurologically healthy screened adult population aged from 40 to 79 years without previous stroke events and recognized CKD and investigated whether a decreased estimated glomerular filtration rate (eGFR) is independently associated with CMB prevalence while considering other known demographic, life styles, medication, and clinical risk factors.

## Materials and methods

### Subjects

The subjects who underwent brain MRI as part of health screening at Seoul National University Hospital Health Promotion Center from January 2009 to December 2013 were included in the study. At the initial visit, they were asked to fill a questionnaire on their sociodemographic status, lifestyle, and medical information. Also, a trained family physician obtained the subjects’ detailed current and past medical histories.

The basic check-up included anthropometric and blood pressure measurements, blood tests after 12-h overnight fasting, urinalysis, electrocardiogram, and basic cancer screening. Optionally, the subjects could choose Brain MRI as an item of health screening.

A total of 2,713 subjects underwent brain MRI during the first period. At first, 15 subjects were excluded since their serum creatinine levels were missing. Then, those aged below 40 years (n = 107) or 80 years or above (n = 15) were excluded. Finally, after excluding 58 subjects with previous symptomatic stroke history, 2,518 subjects were included in this study.

This study was approved by the institutional review board at Seoul National University Hospital (IRB No. 1502-026-647). Informed consent was exempted due to retrospective study design by the institutional review board.

### CMB evaluation

CMBs were defined as well-demarcated focal areas of low signal intensity with associated blooming on the T2-weighted MRI measuring less than 5mm in diameter.[21] The computerized MR images of each subject were independently reviewed and rated. Two reviewers (J-S Lim and H-M Kwon) who were unaware of the subjects’ clinical information counted the CMBs throughout the entire brain. In addition, the locations of CMBs were subdivided into 2 categories: 1) strictly lobar CMBs (located only in cerebral cortex, subcortical junction, and insula), and 2) deep (basal ganglia, thalamus, corona radiata, internal and external capsule) or infratentorial (brainstem and cerebellum) CMBs, with/without lobar CMBs. The previously noted inter-observer reliability coefficient for the presence of CMBs was 0.83.[22] Disagreements were resolved by discussion with a third reviewer. MRI examinations were performed at field strengths of 1.5 T (Signa, GE Healthcare, Milwaukee, WI or Magnetom SONATA, Siemens, Munich, Germany). The imaging protocol consisted of the following: T2-weighted fast spin-echo (repetition time/echo time = 5000/127 ms), T1-weighted spin-echo (repetition time/echo time = 500/11 ms), and fluid-attenuated inversion recovery (repetition time/echo time = 8800/127 ms; inversion time = 2250 ms) imaging. Images were obtained as 26 transaxial slices per scan. The slice thickness was 5 mm with 1-mm interslice gap.

### Renal dysfunction estimated by GFR

GFR was estimated using the Modification of Diet in Renal Disease (MDRD) formula (GFR = 175 × serum creatinine^-1.154^ × age^-0.203^ × 0.742 [for females]).[23] The eGFR was staged according to chronic kidney function categorization of the US National Kidney Foundation (normal (≥90) renal function, mild (60 to 89.9), moderate (30 to 59.9), and severe (<30 mL/min/1.73 m^2^) renal dysfunction).[24]

### Clinical variables

From the items of the health screening program, we identified the possible co-factors associated with the presence of CMB, in light of the findings of previous studies.[13–20, 25, 26] The anthropometric indices included body mass index and waist circumference. After measuring each subject’s weight and height (the subjects were asked to wear a light gown for this purpose), the body mass index was calculated as weight in kilograms divided by the square of the height in meters (kg/m^2^). Also, the waist circumference was measured at the middle point between the lower border of the lowest rib and upper border of iliac crest at the end of a normal expiration. Smoking habit was divided into 2 categories: 1) current smoker and 2) non-, or ex-smoker. Blood pressure was measured after resting for more than 5 minutes in the sitting position. Those who were taking antihypertensive drugs or had high systolic blood pressure (≥140 mmHg) or diastolic blood pressure (≥90 mmHg) were considered as having hypertension. Serum glucose, hemoglobin A1c, triglyceride, low-density lipoprotein cholesterol, high-density lipoprotein cholesterol, total cholesterol, C-reactive protein, and creatinine levels were measured using 12-h fasting blood samples. Subjects who were taking antidiabetic drugs or had high fasting blood glucose (≥126 mg/dL) or hemoglobin A1c levels (≥6.5%) were considered as having diabetes. Dyslipidemia was defined as total cholesterol level of 240 mg/dL or more. Also, those who were taking lipid-lowering drugs were considered as having dyslipidemia. In addition, subjects who were taking drugs which could increase bleeding risk, such as aspirin, clopidogrel, and warfarin, were identified. To identify subjects with atrial fibrillation, a trained physician reviewed each electrocardiogram using the definition of atrial fibrillation from the ACC/AHA/ESC guideline was used.[27]

### Statistical analysis

For each baseline variable, according to the presence of CMB, we performed the *t-*test for continuous variables and chi-square test (or Fisher’s exact test as appropriately) for categorical variables to determine each variable’s crude association with CMB prevalence. *P* for trend was estimated to assess the association between the number of CMB lesions and the stage of renal function.

To evaluate the association between renal dysfunction and presence of CMB, factors with *p* values of less than 0.1 from the univariate analysis were included in the subsequent logistic regression analysis. In addition, demographic characteristics including age and sex were included as co-factors. At first, unadjusted logistic regression was conducted to assess the association between each factor and presence of CMB. Then, multivariate logistic regression analysis was conducted to assess the association between renal dysfunction and presence of CMB after adjusting for those factors. Since there were only 6 subjects with eGFR < 30 (n = 4 for subjects without CMB and n = 2 for subjects with CMB), we used a single category of eGFR < 60 for renal dysfunction group. In addition, the trend of CMB prevalence according to the advancing stages of renal dysfunction was evaluated by using the *p* values from the trend test. As the association between renal dysfunction and presence of CMB might be different according to the CMB location,[28] we performed subgroup analysis stratified by the CMB location.

All statistical analyses were conducted using the STATA software version 14.0 (StataCorp., TX); *p* values lower than 0.05 were considered statistically significant.

## Results

### Baseline characteristics of the study population

The distribution of subjects according to their baseline characteristics and presence of CMB is illustrated in Table 1. The mean age of the overall study population was 57.5 ± 8.3 years (from 40 to 79 years); 1,367 (54.3%) subjects were male. The mean systolic blood pressure and diastolic blood pressure was 125.4 ± 15.2 and 76.7 ± 10.5 mmHg, respectively. The mean serum creatinine level was 0.88 ± 0.21 mg/dL. Most subjects showed normal (26.1%) or mildly impaired (68.5%) renal dysfunction, and the proportion of subjects with moderate (5.2%) or severe (0.2%) renal dysfunction was very low.

**Table 1:** Baseline characteristics of the study population.

			Presence of CMB					
	Total (*N* = 2,518)		Yes (*N* = 103)		No (*N* = 2,415)		*P* value*	
Age—year	57.5	± 8.3	61.1	± 8.7	57.3	± 8.3	<	0.001
Sex—no. (%)								
Male	1,367	(54.3)	63	(61.2)	1,304	(54.0)		0.153
Female	1,151	(45.7)	40	(38.8)	1,111	(46.0)		
Smoking- no. (%)								
Non- or Ex-smoker	2,100	(83.4)	90	(87.4)	2,010	(83.2)		0.268
Current Smoker	418	(16.6)	13	(12.6)	405	(16.8)		
Hypertension^†^ - no. (%)								
No	1,561	(62.0)	47	(45.6)	1,514	(62.7)	<	0.001
Yes	957	(38.0)	56	(54.4)	901	(37.3)		
Diabetes^‡^ - no. (%)								
No	2,108	(83.7)	76	(73.8)	2,032	(84.1)		0.005
Yes	410	(16.3)	27	(26.2)	383	(15.9)		
Dyslipidemia^§^ - no. (%)								
No	1,931	(76.7)	79	(76.7)	1,852	(76.7)		0.998
Yes	587	(23.3)	24	(23.3)	563	(23.3)		
Anticoagulation or Anti-platelet Therapy^∥^- no. (%)								
cNo	2,162	(85.9)	79	(76.7)	2,083	(86.3)		0.006
Yes	356	(14.1)	24	(23.3)	332	(13.8)		
Atrial Fibrillation- no. (%)								
No	2,502	(99.4)	103	(100.0)	2,399	(99.4)		1.000**
Yes	14	(0.6)	0	(0.0)	14	(0.6)		
Silent Lacunar Infarction- no. (%)								
No	2,355	(93.5)	81	(78.6)	2,274	94.2	<	0.001
Yes	163	(6.5)	22	(21.4)	141	5.8		
Body Mass Index—kg/m^2^	24.2	± 3.0	24.0	± 3.2	24.2	± 3.0		0.647
Waist Circumference—cm	86.2	± 8.9	86.9	± 9.3	86.2	± 8.9		0.400
Systolic Blood Pressure—mmHg	125.4	± 15.2	129.9	± 17.3	125.2	± 15.0		0.002
Diastolic Blood Pressure—mmHg	76.7	± 10.5	77.0	± 11.7	75.6	± 10.5		0.183
Creatinine—mg/dL	0.88	± 0.21	0.96	± 0.41	0.88	± 0.20	<	0.001
eGFR^¶^ - ml/min/1.73 m^2^	81.5	± 15.5	78.4	± 20.8	81.6	± 15.2		0.039
≥ 90 ml/min/1.73 m^2^—no. (%)	657	(26.1)	21	(20.4)	636	(26.3)	<	0.001**
60–89.9 ml/min/1.73 m^2^—no. (%)	1,725	(68.5)	66	(64.1)	1,659	(68.7)		
30–59.9 ml/min/1.73 m2—no. (%^)^	130	(5.2)	14	(13.6)	116	(4.8)		
< 30 ml/min/1.73 m2—no. (%)	6	(0.2)	2	(1.9)	4	(0.2)		
Total Cholesterol—mg/dL	199.8	± 36.3	198.5	± 34.0	199.8	± 36.4		0.722
LDL—mg/dL	127.0	± 34.8	127.3	± 32.8	127.0	± 34.9		0.913
HDL—mg/dL	54.8	± 14.1	53.1	± 13.5	54.8	± 14.1		0.218
TG—mg/dL	115.4	± 67.6	110.9	± 57.9	115.6	± 68.0		0.494
CRP—mg/dL	0.18	± 0.68	0.15	± 0.35	0.18	± 0.69		0.688
Fasting Blood Glucose—mg/dL	97.4	± 23.6	99.9	± 24.7	97.3	± 23.6		0.289
HbA1c - %	5.91	± 0.82	6.06	± 0.94	5.90	± 0.81		0.055

CMB, cerebral microbleed; SD, standard deviation; eGFR, estimated glomerular filtration rate; LDL, low density lipoprotein; HDL, high density lipoprotein; TG, triglyceride; CRP, C-reactive protein; HbA1c, hemoglobin A1c.

Plus–minus values are means ± SD. Comparison was performed between with and without CMB groups.

There were some missing values (2 for atrial fibrillation, 9 for waist circumference, 22 for LDL, 13 for HbA1c, 8 for CRP, and 7 for HDL and TG respectively).

* t-test for continuous variables and chi-square test for categorical variables.

† Those who were taking antihypertensive drugs or SBP ≥ 140 mmHg, or DBP ≥ 90 mmHg.

‡ Those who were taking antidiabetic drugs or fasting blood glucose ≥ 126 mg/dL or Hb A1c ≥ 6.5%.

§ Those who were taking lipid lowering drugs or total cholesterol ≥ 240 mg/dL.

∥Those who were taking aspirin, clopidogrel, warfarin, or other antiplatelet drugs.

¶ Calculated using the Modification of Diet in Renal Disease (MDRD) formula.

** Fisher's exact test.

The prevalence of CMB in all participants was 4.1% (n = 103), with 55.3% (n = 57) of CMBs located in the deep or infratentorial regions. Subjects with CMB were significantly older (61.1 ± 8.7 years) than those without CMB (57.3 ± 8.3 years; *p* < 0.001). Subjects with CMB showed a higher prevalence of moderate-to-severe renal dysfunction (15.5% vs. 5.0%; *p* < 0.001). They also showed a higher proportion of CMB-related risk factors such as hypertension, diabetes, and taking anticoagulation or anti-platelet medication; higher systolic blood pressure; and higher serum creatinine level (all *p* < 0.05).

### Association of renal dysfunction with presence of CMB

As the stage of kidney dysfunction advanced, a significant trend of increase in CMB lesion number was observed (*p* for trend = 0.003) (Table 2).

**Table 2:** The number of cerebral microbleeds and renal function.

	The Number of Cerebral Microbleeds—no. (%)						
	0		1		≥ 2		*P* for Trend*
eGFR—ml/min/1.73 m^2^							
≥ 90	636	(26.3)	17	(21.8)	4	(16.0)	0.003
60–89.9	1,659	(68.7)	51	(65.4)	15	(60.0)	
30–59.9	116	(4.8)	10	(12.8)	4	(16.0)	
< 30	4	(0.2)	0	(0.0)	2	(8.0)	
Total	2,415	(100.0)	78	(100.0)	25	(100.0)	

eGFR, estimated glomerular filtration rate.

* P for trend was calculated by linear regression analysis adjusted for age, sex, diabetes, systolic blood pressure, and anticoagulation or anti-platelet therapy.

As described in the statistical analysis session, factors with *p* values lower than 0.1 were included in the subsequent logistic regression model. Considering the possible multicollinearity between the variables “hypertension” and “systolic blood pressure”, systolic blood pressure was selected since its *p* value was lower than that of hypertension. Age, systolic blood pressure, stage of renal dysfunction, diabetes, and anticoagulation or anti-platelet medication were included in the final logistic regression analysis.

In the unadjusted model, all of the included factors were significantly associated with the presence of CMB. Subjects with moderate-to-severe eGFR decrease were more likely to have CMB (odds ratio (OR), 4.04; 95% Confidence interval (CI), 2.05–7.96). Also, age (*p* < 0.001), systolic blood pressure (*p =* 0.002), diabetes (*p* = 0.006), and taking anticoagulation or anti-platelet medication (*p* = 0.007) were significantly associated with the presence of CMB.

In the multivariate logistic regression model, subjects with moderate-to-severe eGFR decrease were more likely to have CMB, independent of age, systolic blood pressure, diabetes, and taking anticoagulation or anti-platelet medication. The estimated OR was 2.63 (95% CI, 1.35–5.55) for subjects with moderate-to-severe eGFR decrease. Also, age (*p* = 0.003) and systolic blood pressure (*p* = 0.018) were significant independent factors associated with the presence of CMB. In addition, decreasing GFR level was associated with an increasing trend of the presence of CMB in both the unadjusted and adjusted models (*p* for trend = 0.002 and 0.031, respectively) (Table 3).

**Table 3:** Association of renal function with cerebral microbleed (Total *N* = 2,518).

	Unadjusted Model			Adjusted Model (Overall)		
	OR (95% CI)		*P* value	OR (95% CI)		*P* value
eGFR—ml/min/1.73 m^2^						
≥ 90 (reference)						
60–89.9	1.20	(0.73–1.99)	0.465	1.11	(0.67–1.84)	0.697
< 60	4.04	(2.05–7.96)	< 0.001	2.63	(1.29–5.35)	0.008
*P* for Trend			0.002			0.031
Diabetes	1.88	(1.20–2.96)	0.006	1.32	(0.82–2.12)	0.253
Anticoagulation/Anti-platelet Therapy	1.91	(1.19–3.05)	0.007	1.38	(0.84–2.25)	0.205
Female (vs Male)	0.75	(0.50–1.12)	0.154	0.80	(0.53–1.22)	0.300
Age—year	1.05	(1.03–1.08)	< 0.001	1.04	(1.01–1.07)	0.003
Systolic Blood Pressure	1.02	(1.01–1.03)	0.002	1.02	(1.00–1.03)	0.018

OR, odds ratio; CI, confidence interval; eGFR, estimated glomerular filtration rate.

In the adjusted model, data were adjusted for eGFR, age, sex, diabetes, systolic blood pressure, and anticoagulation or anti-platelet therapy.

In the subgroup analysis, moderate-to-severely decreased eGFR levels were significantly associated with deep/infratentorial CMB (OR, 3.11; 95% CI, 1.24–7.79). Although the subjects with moderate-to-severely decreased eGFR levels were more likely to have strictly lobar CMB (OR = 1.90), the association was not statistically significant (95% CI, 0.65–5.56, *p* = 0.240) (Table 4).

**Table 4:** Associations of renal function with cerebral microbleed by the location (Total *N* = 2,518).

	Strictly Lobar (CMB *N* = 46)			Deep/Infratentorial (CMB *N* = 57)		
	OR (95% CI)		*P* value	OR (95% CI)		*P* value
eGFR—ml/min/1.73 m^2^						
≥90 (reference)						
60–89.9	1.04	(0.50–2.16)	0.917	1.16	(0.58–2.32)	0.669
< 60	1.90	(0.65–5.56)	0.240	3.11	(1.24–7.79)	0.015
Diabetes	1.14	(0.56–2.33)	0.725	1.45	(0.78–2.70)	0.235
Anticoagulation or Anti-platelet Therapy	1.62	(0.81–3.25)	0.175	1.17	(0.60–2.30)	0.647
Female (vs Male)	0.88	(0.48–1.61)	0.680	0.75	(0.43–1.30)	0.307
Age—year	1.05	(1.01–1.08)	0.019	1.03	(1.00–1.07)	0.060
Systolic Blood Pressure	1.01	(0.99–1.03)	0.241	1.02	(1.00–1.04)	0.037

CMB, cerebral microbleed; OR, odds ratio; CI, confidence interval; eGFR, estimated glomerular filtration rate.

Adjusted for eGFR, age, sex, diabetes, systolic blood pressure, and anticoagulation or anti-platelet therapy.

As it is possible that other cerebral small vessel disease markers could influence the relationship between CMBs and renal dysfunction, we repeated the multivariate logistic regression analysis while controlling for Silent lacunar infarction (SLI) status. SLI was defined as a focal infarction with a fluid-filled cavity (signal similar to the cerebrospinal fluid signal), 3 mm to 15 mm in diameter within subcortical regions.[21] The positive association between moderate-to-severely decreased eGFR levels and CMBs remained significant (OR, 2.48; 95% CI, 1.21–5.07) (S1 File).

## Discussion

In this study, we investigated the association between renal dysfunction, according to eGFR, and the presence of CMB and the number of CMB lesions using detailed anthropometric, lifestyle, and clinical features of neurologically healthy Korean adults without stroke and known chronic kidney disease history. We found that renal dysfunction was significantly associated with the presence of CMB and the number of CMB lesions, independent of other known CMB risk factors in a neurologically healthy population without perceived chronic kidney disease or previous stroke history. In addition, the association was more evident for deep or infratentorial CMB than for the strictly lobar CMB.

A few studies investigated the association between renal dysfunction and the presence of CMB.[17–20] As mentioned above, these studies had important limitations including a small sample size and study population with specific diseases such as stroke[17, 18, 20] or advanced kidney disease (mean serum creatinine level = 2.91 without CMB, and 5.03 with CMB, respectively),[19] thus hindering the generalizability of the results. Furthermore, the baseline characteristics of the study population might have been different from those of the general population and hospitalized patients. When we compared the results between the study population and the general population, the difference resulted in a higher prevalence of risk factors for CMB in hospitalized patients, such as hypertension.[17, 18]

Recently, Akoudad et al reported that there was no significant association between renal dysfunction assessed by eGFR level and the presence of CMB.[28] Although the study included, in general, healthy subjects like those in our study, the discrepant result can potentially be explained by the differences in the prevalence of CMB and ethnicity.[29, 30] The prevalence of CMB in present study (4.1%) was around the previously noted prevalence in asymptomatic population. However, the prevalence of CMB in Akoudad’s study (12.6%) was substantially higher.[2, 5, 31] Furthermore, in contrast to Western populations, Asian populations are more likely to have hypertensive cerebral microangiopathy, rather than cerebral amyloid angiopathy. While hypertensive cerebral microangiopathy may be attributed to the same pathophysiologic mechanism as renal dysfunction, cerebral amyloid angiopathy affects only the brain.[15, 25, 29] In general, strictly lobar CMB is known to be caused by cerebral amyloid angiopathy, and deep CMB is known to be caused by hypertensive microangiopathy.[25] Accordingly, most CMBs (73.3%) included in Akoudad’s study were located strictly in the lobar regions and more than half of CMBs in the present study were located in the deep or infratentorial regions. As the proportion of subjects with deep or infratentorial CMB was higher in our study, the association between renal dysfunction and CMB might be more evident in our study.[15, 28, 29] This finding also supports the assumption that the association between renal dysfunction and overt CVD can be different across diverse ethnic groups.[29, 30]

In the present study, a large sample size of a neurologically healthy population with normal biochemical and metabolic profiles, including mean serum creatinine level and blood pressure, might allow the results to be generalized to the Korean population. This enabled us to investigate the association between kidney dysfunction and presence of CMB more closely. In addition, as the present study involved subjects who visited the clinic for health screening, various possible co-factors such as anthropometric, biochemical, and metabolic profiles could be considered. Furthermore, most of the baseline characteristics including body mass index, waist circumference, blood pressure, and serum creatinine level were within the normal range. Also, we included young adults in their 40 s (n = 457, 18.1%) and 50 s (n = 1,129, 44.8%) as well as the elderly population. All of these points are the strengths of the present study that highly support the generalizability of the results.

In this study, renal dysfunction was significantly associated with the presence of CMB. As the stage of kidney dysfunction advanced, the estimated OR for the presence of CMB increased. Also, the association was consistent after adjusting for significant co-factors. Moreover, there was an increasing trend of the presence of CMB as the GFR decreased. The number of CMB lesions was higher in those with than in those without advanced renal dysfunction, suggesting a dose-response relationship.[17] Although previous studies which dealt with renal dysfunction and CMB used different population groups such as those with overt CVD or chronic renal disease, the association between renal dysfunction and presence of CMB was consistent across those studies.[17–20] The association was significant even in neurologically healthy subjects in the present study. As CMB is generally considered an independent risk factor of overt CVD,[1–3] these findings highlight the possibility of renal function being a good surrogate marker of cerebrovascular events.[17, 20]

Cho et al. suggested 2 possible mechanisms of the association between renal dysfunction and CMB.[20] First, the microvascular beds of both the kidney and brain are similar and susceptive to hypertensive manifestation. Therefore, intercurrent damages in both end organs could be the result of the same causal mechanism such as hypertensive microvascular damage. Second, as the association between renal dysfunction and CMB is independent of other risk factors, renal dysfunction can be an independent risk factor for CMB. Increased inflammation and endothelial dysfunction in chronic kidney disease may affect the brain’s microvascular system. In the present study, renal dysfunction as well as systolic blood pressure was associated with the presence of CMB independently. This increases the likelihood of renal dysfunction being a risk factor, independent of blood pressure. However, it is still unclear whether renal dysfunction and CMB are independent synchronous end organ damages caused by hypertension or whether renal dysfunction is an independent risk factor of CMB since our study was a cross-sectional study. Although increased inflammation in renal dysfunction could be a possible underlying mechanism, the association between CRP level and presence of CMB was not significant in this study (observed using a dummy variable of CRP level with various cutoffs) (S2 File). Further studies are needed to elucidate the underlying mechanism of CMB in subjects with renal dysfunction.

In multivariate analysis, age, systolic blood pressure, and renal dysfunction were significantly associated with presence of CMB. In addition, diabetes and the use of anticoagulation or anti-platelet therapy were not associated with the presence of CMB. The findings are consistent with previous studies regarding renal dysfunction and CMB.[18–20] Considering both previous studies and our study, blood pressure management can be seen as useful not only for kidney protection[32] but also for CVD prevention in those with renal dysfunction. Except for blood pressure, there are controversies regarding the factors associated with CMB. Although several studies have indicated a significant association between CMB and anticoagulation or anti-platelet therapy,[33–35] no significant association was found in this study. Also, some studies had reported no significant association between CMB and those drugs.[18, 19, 36] In addition, in some previous studies, there was a significant association between the presence of CMB and other factors including sex and smoking in the general population.[37–39] Although these discordances could be explained by the differences in study design, ethnicity, and baseline characteristics, further investigations are needed to verify these associations.

There are some limitations to this study. First, those subjects who underwent brain MRI as part of their general health check-up might have chosen the expensive brain MRI since they had some risk factors or related symptoms of stroke. Therefore, the results may be different from those of the general population. However, the prevalence of CMB in the general population in this study was within the range of the prevalence (3 to 6%) reported in previous studies.[2, 37–39] Also, we considered several risk factors, and they were included in the statistical model. Second, the results of the present study were obtained from a cross-sectional analysis that precludes establishment of a causal relationship between renal dysfunction and CMB. A prospective study with multi-center involvement is therefore recommended to determine whether there is a causal relationship between renal dysfunction and CMB. Finally, as the present study included subjects without perceived chronic kidney disease, the number of subjects with moderate-to-severe renal dysfunction was relatively small. Only 4 subjects had severe renal dysfunction.

While subjects with both moderate (OR, 2.50; 95% CI, 1.21–5.20) and severe (OR, 8.55; 95% CI, 1.44–50.65) dysfunction had significant association with the presence of CMB (S3 File). A study with a larger sample size may be necessary to elucidate the risk of CMB according to each renal function category.

In conclusion, we found a significant association between renal dysfunction and presence of CMB in neurologically healthy adults. As CMB is one of the well-known risk factors of overt CVD, [1–3, 5] renal function can be a useful marker of cerebrovascular events.[17, 20] More studies are needed to evaluate if treatment of kidney disease and risk factor modification may prevent further progress of CMB.

## Supporting information

S1 File Analysis Including Silent Lacunar Infarction as a Co-factor. (DOCX)

S2 File C-reactive Protein Level and Presence of CMB. (DOCX)

S3 File Association of Each Renal Function Category with Cerebral Microbleed (Total *N* = 2,518). (DOCX)
