# Correction: Kidney dysfunction and cerebral microbleeds in neurologically healthy adults

**DOI:** 10.1371/journal.pone.0176901

**Published:** 2017-04-27

**Authors:** Sang Hyuck Kim, Dong Wook Shin, Jae Moon Yun, Ji Eun Lee, Jae-Sung Lim, Be Long Cho, Hyung-Min Kwon, Jin-Ho Park

Additional affiliations for the sixth, seventh, and last authors are not indicated. Be Long Cho and Jin-Ho Park are also affiliated with: Department of Family Medicine, College of Medicine, Seoul National University, Seoul, Republic of Korea. Hyung-Min Kwon is also affiliated with: Department of Neurology, College of Medicine, Seoul National University, Seoul, Republic of Korea.
