# Supplementary material for: Kidney dysfunction and cerebral microbleeds in neurologically healthy adults
Source: PLoS One. 2017 Feb 16;12(2):e0172210. doi: 10.1371/journal.pone.0172210 (PMC5312922; doi:10.1371/journal.pone.0172210)
Supplement: S1 File — (DOCX) [file pone.0172210.s001.docx]

| **S1 Table. Analysis Including Silent Lacunar Infarction as a Co-factor.** | | | |
| --- | --- | --- | --- |
|  | Adjusted Model | | |
|  | OR (95 % CI) | | *P* value |
| eGFR - ml/min/1.73 m^2^ |  |  |  |
| ≥ 90 (reference) |  |  |  |
| 60-89.9 | 1.12 | (0.67 - 1.86) | 0.664 |
| < 60 | 2.48 | (1.21 - 5.07) | 0.013 |
| *P* for Trend |  |  | 0.041 |
| Diabetes | 1.25 | (0.78 - 2.03) | 0.353 |
| Anticoagulation/Anti-platelet Therapy | 1.40 | (0.85 - 2.29) | 0.184 |
| Female (vs Male) | 0.80 | (0.53 - 1.21) | 0.295 |
| Age - year | 1.03 | (1.00 - 1.06) | 0.028 |
| Systolic Blood Pressure | 1.01 | (1.00 - 1.03) | 0.030 |
| Silent Lacunar Infarction | 3.00 | (1.76 - 5.10) | < 0.001 |

OR, odds ratio; CI, confidence interval ; eGFR, estimated glomerular filtration rate

In the adjusted model, data were adjusted for eGFR, age, diabetes, systolic blood pressure, anticoagulation or anti-platelet therapy, and presence of silent lacunar infarction
