# Supplementary material for: Kidney dysfunction and cerebral microbleeds in neurologically healthy adults
Source: PLoS One. 2017 Feb 16;12(2):e0172210. doi: 10.1371/journal.pone.0172210 (PMC5312922; doi:10.1371/journal.pone.0172210)
Supplement: S2 File — (DOCX) [file pone.0172210.s002.docx]

| **S2 Table. C-reactive Protein Level and Presence of CMB** | | | | | | | | |
| --- | --- | --- | --- | --- | --- | --- | --- | --- |
|  |  |  | Presence of CMB | | | |  |  |
|  | Total (*N* = 2,510) | | Yes (*N* = 102) | | No (*N* = 2,408) | | *P* value^*^ | |
| CRP - mg/dL | 0.18 | ± 0.68 | 0.15 | ± 0.35 | 0.18 | ± 0.69 |  | 0.688 |
|  |  |  |  |  |  |  |  |  |
| CRP Groups |  |  |  |  |  |  |  |  |
| < 0.1 | 1,570 | (62.6) | 60 | (58.8) | 1,510 | (62.7) |  | 0.427 |
| ≥0.1 | 940 | (37.5) | 42 | (41.2) | 898 | (37.3) |  |  |
|  |  |  |  |  |  |  |  |  |
| < 0.3 | 2,240 | (89.2) | 89 | (87.3) | 2,151 | (89.3) |  | 0.508 |
| ≥0.3 | 270 | (10.8) | 13 | (12.8) | 257 | (10.7) |  |  |
|  |  |  |  |  |  |  |  |  |
| < 0.5 | 2,366 | (94.3) | 97 | (95.1) | 2,269 | (94.2) |  | 0.711 |
| ≥0.5 | 144 | (5.7) | 5 | (4.9) | 139 | (5.8) |  |  |
| CMB, cerebral microbleed; CRP, C-reactive protein | | | | |  |  |  |  |
| * t-test for continuous variables and chi-square test for categorical variables | | | | | | |  |  |
| 8 subjects from original 2,518 subjects were excluded due to missing in CRP levels | | | | | | |  |  |
