# Supplementary material for: Kidney dysfunction and cerebral microbleeds in neurologically healthy adults
Source: PLoS One. 2017 Feb 16;12(2):e0172210. doi: 10.1371/journal.pone.0172210 (PMC5312922; doi:10.1371/journal.pone.0172210)
Supplement: S3 File — (DOCX) [file pone.0172210.s003.docx]

|  |
| --- |

| **S3 Table. Association of Each Renal Function Category with Cerebral Microbleed (Total *N* = 2,518)** | | | | | | | |
| --- | --- | --- | --- | --- | --- | --- | --- |
|  | Unadjusted Model | | |  | Adjusted Model (Overall) | | |
|  | OR (95 % CI) | | *P* value |  | OR (95 % CI) | | *P* value |
| eGFR - ml/min/1.73 m^2^ |  |  |  |  |  |  |  |
| ≥ 90 (reference) |  |  |  |  |  |  |  |
| 60-89.9 | 1.20 | (0.73 - 1.99) | 0.465 |  | 1.11 | (0.67 - 1.84) | 0.700 |
| 30-59.9 | 3.66 | (1.81 - 7.39) | < 0.001 |  | 2.40 | (1.15 - 5.01) | 0.019 |
| < 30 | 15.14 | (2.63 - 87.33) | 0.002 |  | 8.20 | (1.38 - 48.64) | 0.021 |
| *P* for Trend |  |  | 0.001 |  |  |  | 0.016 |
| Diabetes | 1.88 | (1.20 - 2.96) | 0.006 |  | 1.30 | (0.80 - 2.09) | 0.288 |
| Anticoagulation/Anti-platelet Therapy | 1.91 | (1.19 - 3.05) | 0.007 |  | 1.39 | (0.85 - 2.28) | 0.190 |
| Female (vs Male) | 0.75 | (0.50 - 1.12) | 0.154 |  | 0.80 | (0.53 - 1.22) | 0.301 |
| Age - year | 1.05 | (1.03 - 1.08) | < 0.001 |  | 1.04 | (1.01 - 1.07) | 0.003 |
| Systolic Blood Pressure | 1.02 | (1.01 -1.03) | 0.002 |  | 1.02 | (1.00 - 1.03) | 0.020 |
| OR, odds ratio; CI, confidence interval; eGFR, estimated glomerular filtration rate | | | | | | | |
| In the adjusted model, data were adjusted for eGFR, age, sex, diabetes, systolic blood pressure, and anticoagulation or anti-platelet therapy | | | | | | | |
|  |  |  |  |  |  |  |  |
